# Clinical significance of trisomy 8 that emerges during therapy in chronic myeloid leukemia

**DOI:** 10.1038/bcj.2016.96

**Published:** 2016-11-04

**Authors:** W Wang, Z Chen, Z Hu, C C Yin, S Li, S Bai, C E Bueso-Ramos, L J Medeiros, S Hu

**Affiliations:** 1 Department of Hematopathology, The University of Texas MD Anderson Cancer Center, Houston, TX, USA

Chronic myeloid leukemia (CML) is defined by t(9;22)(q34;q11), a genetic abnormality producing *BCR-ABL1* fusion on derivative chromosome 22.^1^ At cytogenetic level, t(9;22)(q34;q11) is the sole abnormality in over 90% of patients in chronic phase (CP). As the disease progresses to accelerated phase (AP) and blast phase (BP), clonal evolution occurs commonly with the emergence of additional cytogenetic abnormalities (ACAs). Approximately 30% of patients with CML-AP and 70–80% of patients with CML-BP have ACAs.^2, 3, 4^ Among various ACAs, trisomy 8 (+8) and an extra copy of philadelphia chromosome (Ph) are most common.^5, 6^ Different ACAs have been shown to be associated with different impact on treatment response and survival. Some ACAs are associated with disease progression and treatment resistance, whereas others may simply reflect the genetic instability induced by continuous activation of *BCR-ABL1*.^6, 7, 8^ In addition, the interval from initial diagnosis of CML to emergence of ACAs may also be important as the same ACA can have differential impact on tyrosine kinase inhibitor (TKI) treatment and prognosis when it emerges at the time of initial CML diagnosis versus during therapy.^6^ In the recently updated World Health Organization (WHO) criteria for AP of CML, any new clonal chromosomal abnormality in t(9;22) positive cells that occurs during therapy is considered as a criterion for AP.^9^

In an earlier study,^10^ we showed that +8 occurred in ~30% of CML patients who developed ACAs and it often coexisted with other ACAs. When focusing on patients with isolated +8 (no other concurrent ACAs), we found that +8 was associated with a relatively better prognosis than other ACAs except -Y. However, when compared with patients without any ACAs, patients with +8 as a group had a worse survival. In that study, however, we did not stratify cases based on the time of +8 emergence (at CML diagnosis versus during therapy) or whether there were other concurrent AP features. In the following study, we stratified cases with isolated +8 based on the time of +8 emergence and found that there was no adverse survival impact when +8 was present at the time of initial CML diagnosis, whereas +8 was associated with a poorer prognosis when it emerged during therapy.^6^ In patients with +8 emerging during therapy, we noticed that some patients also had other concurrent accelerated features, such as increased blasts. Thus, the poorer survival associated with +8 emerging during therapy might be attributable to those concurrent accelerated features rather than +8. As mentioned above, in the 2016 updated WHO classification, any new clonal chromosomal abnormalities that emerge during therapy are considered as a criterion of AP. This criterion triggered us to perform this study with an aim to explore whether the emergence of +8 during therapy is a feature of AP that causes treatment resistance and disease progression. We focused on patients with isolated +8 emerging during therapy, excluding cases with simultaneous presence of other AP features (such as increased blasts), to explore the exact role of +8 in CML. We studied the dynamic course of +8, its association with t(9;22) and its impact on treatment response and survival.

We focus on the karyotype^11^ at the time ACAs initially emerge. Cases with inadequate clinical information were excluded. In 2013 CML patients, 37 patients developed +8 as the sole additional chromosomal abnormality. Of note, cases with +8 in Ph-negative cells are not considered as ACAs and thus were not included. A summary of these 37 patients is illustrated in Figure 1a. In detail, 6 patients had +8 at the time of initial CML diagnosis and 31 patients developed +8 during therapy. Among these 31 patients with +8 emerging during therapy, 28 had +8 as the only feature of disease acceleration, whereas the remaining 3 had other concurrent features of disease progression, manifested as increased blasts (30, 22 and 76% blasts, respectively). To eliminate the confounding impact of increased blasts, we excluded these 3 patients and focused on the 28 patients who had +8 only without any other features of AP. Their karyotype, the course of treatment, treatment response and survival are summarized in Table 1.

There were 15 men and 13 women with a median age of 44 (range, 20–71 years) at the time of CML diagnosis. After a median interval of 29 months (range, 3–98 months), patients developed +8 in Ph-positive cells. The median percentage of metaphases that had +8 was 22.5% (range, 7–75%). We next reviewed the karyotype immediately before +8 emergence, which was performed at a median of 3.6 months (range 1.3–37.8 months) prior to +8 emergence. Among these 28 cases, 24 (86%) showed positive t(9;22). In the remaining 4 cases (cases #11, 14, 18 and 23), although conventional cytogenetic analysis did not reveal t(9;22) prior to the emergence of +8, molecular studies were all positive for *BCR-ABL1* fusion transcripts and the percentages of *BCR-ABL1* to *ABL1* transcripts were 13.4, 8.8, 70 and 7.6, respectively. Of note, in cases #11, 14 and 23, molecular studies were performed at the same time of karyotyping analysis, whereas in case #18, in which molecular study showed *BCR-ABL1/ABL1* of 70% and karyotyping showed no t(9;22), molecular study was performed 4.5 months prior to the +8 emergence and karyotyping was performed 24.3 months prior to the +8 emergence.

The therapeutic regimens before and after +8 emergence are listed in Table 1. Two patients (case #27 and 28) lacked detailed clinical information about treatment after +8 emergence. In the remaining 26 patients, 24 (92%) received TKI therapy after the emergence of +8, and the remaining 2 patients (cases #6 and 25) did not receive TKIs due to prior TKIs' resistance or toxicity; both underwent stem cell transplant. In total, 8 of 26 (31%) patients underwent stem cell transplant (Table 1). For treatment response, 21 patients had adequate clinical follow-up for evaluating response and they can be divided into two groups. Group 1 had 15 (71%) patients who achieved complete cytogenetic response (CCyR) and major molecular response (MMR). These patients also showed the disappearance of +8 clones. Interestingly, 5 patients (case #1, 2, 3, 14 and 23) in this group showed the disappearance of +8 occurred before the disappearance of t(9;22). The dynamic change of +8 and t(9;22) from a representative patient (case #2) is illustrated in Figure 1b. Group 2 had 6 (29%) patients (case #4, 10, 12, 15, 20 and 24) who did not achieve CCyR. Although these patients had persistent t(9;22), all showed the disappearance of +8 at some time-point after therapy (Table 1). A representative case (case #4) is illustrated in Figure 1c. The persistence of t(9;22) and disappearance of +8 indicates that +8 did not play a role in mediating resistance to TKIs treatment in these 6 patients.

Three (case #10, 12 and 21) patients developed blastic transformation. In cases #10 and #12, 100% of metaphases had t(9;22) at the time of BP, whereas only 10% of metaphases in case #10 and no metaphases in case #12 had +8. This indicates that +8 likely does not have an important role in inducing blast transformation. Conventional karyotypic analysis was not performed in case #21 at the time of blastic transformation, thus the status of +8 is unknown.

The median follow-up is 65 months (range, 4–200 months), calculated from the time of +8 emergence. At the last follow-up, 93% (14/15) patients who achieved CCyR and MMR were alive, whereas only 15% (1/6) patients who did not achieved CCyR and MMR were alive (15 versus 93%, *P*=0.0017, Fisher's exact test, two-tailed); the only patient (case #20) who did not achieve CCyR and MMR but was alive achieved partial cytogenetic response with only 5% metaphases positive for t(9;22) at the last follow-up. When compared with patients with no ACAs, patients with +8 showed no significant difference in overall survival, although there is a trend toward worse survival in patients with +8 (Figure 1d).

It is of interest that the size of +8 clones was variable at the time of its emergence (7% to 75%), which triggers us to examine whether the size of +8 clones is associated with different treatment response and survival. We divided the cases into two groups: Group A (14 cases) with ⩽20% cells having +8, and Group B (14 cases) with >20% having +8. First, we analyze the treatment response. In Group A, 13 patients had enough cytogenetic follow-up and CCyR/MMR is 62% (8/13). In Group B, 8 patients had enough cytogenetic follow-up and CCyR/MMR is 87.5% (7/8). There was no significant difference between these two groups on treatment response (*P*=0.34, Fisher's exact test, two tailed). Next, we analyze patients' survival. Similar to the treatment response, there was no survival difference between these two groups (*P*=0.85) (Figure 1e).

In summary, we analyzed CML patients who developed +8 during therapy. We excluded patients with other confounding factors, such as other concurrent ACAs or other features of AP. We found that +8 often arose from a background of positive t(9;22). The percentage of metaphases with +8 was relatively low (22.5%) at the time of its emergence. In all patients with adequate cytogenetic follow-up, +8 disappeared at some time-point after therapy, even in those patients who did not achieved CCyR with persistent t(9;22) (Figure 1c). The overall treatment response was favorable. Although there was a trend of worse prognosis in patients with +8, the difference was not statistically significant (Figure 1d). In addition, the size of +8 clone had no effect on treatment response and survival (Figure 1e). These results suggest that the emergence of +8 during therapy does not have a critical role in inducing treatment resistance and disease progression. We therefore propose that, similar to isolated +8 identified at initial CML diagnosis,^6^ the emergence of +8 during therapy should not be used as a criterion for AP either. The emergence of isolated +8 in CML patients may solely be a secondary event that reflects the overall genetic instability induced by the persistent *BCR-ABL1*. The relatively worse prognosis associated with +8 presented in previous studies is likely attributable to the concurrent presence of other ACAs or other AP features.^10, 12, 13, 14^ This is different from some other cytogenetic abnormalities, such as 3q26.2 rearrangements, i(17)(q10), and -7/del7q. These abnormalities can trigger and drive disease progression and treatment resistance in CML. Of note, although our CML cohort is large and composed of 2013 patients, the number of patients included in our study is relatively small (28 in total) and further study with more cases will be appropriate to elucidate the exact role of +8 in CML.

## Figures and Tables

**Figure 1:**
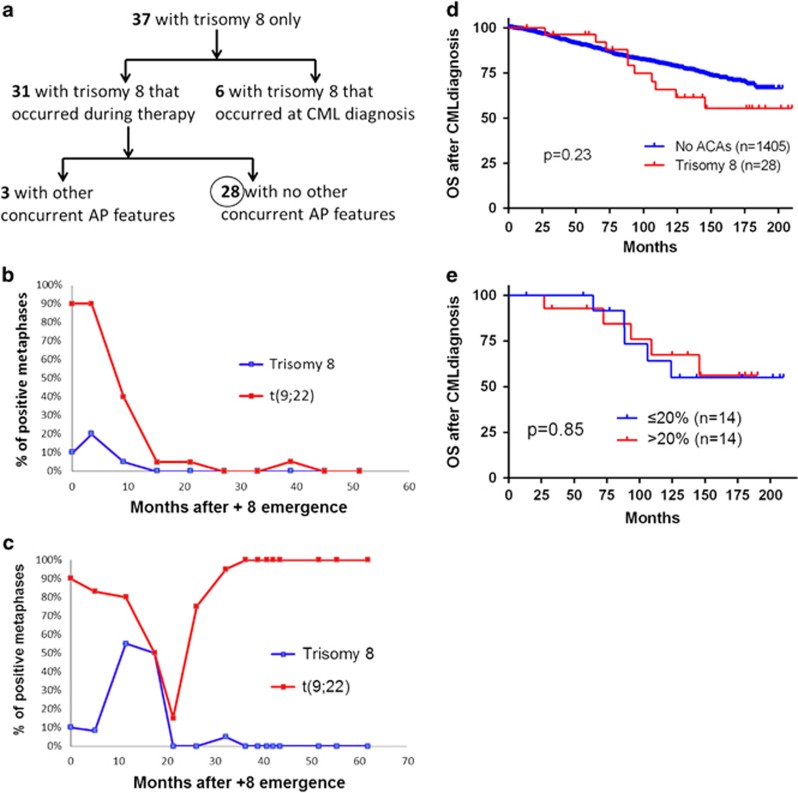
(**a**) Summary of CML patients with isolated +8 (no other concurrent ACAs) in our CML cohort. The 28 patients with +8 emerging during therapy without other concurrent AP features were included in this study. (**b**) Percentages of metaphases containing +8 (blue) and t(9;22) (red) during the course of CML treatment in case #2. +8 disappeared at 15 months after its emergence, and t(9;22) disappeared later (27 months) with a transient re-emergence at 39 months. (**c**) Percentages of metaphases containing +8 (blue) and t(9;22) (red) during the course of CML treatment in patient #4. +8 disappeared at 21 months after its emergence with a transient re-emergence at 32 months, whereas t(9;22) persisted during the whole course of therapy. (**d**) Survival comparison between patients with isolated +8 and patients with no any ACAs. The Kaplan and Meier method was used to build survival curves and the log-rank test was performed to evaluate the differences in survival. The survival is calculated from the time of CML diagnosis to the last follow-up. (**e**) Survival comparison between patients with ⩽20% +8 clones and patients with >20% +8 clones.

**Table 1:** Clinicopathological characteristics of CML patients with +8 only emerging during therapy

	*Age/sex*	*Karyotype*	*Before*	*Interval-1*	*After*	*CCyR*	*MMR*	*Disappearance of +8*	*BP*	*Interval-2*	*Status*
#1	56/M	A: 46,XY,t(9;22)(q34;q11)[20] B: 46,XY,t(9;22)(q34;q11)[23]/47,XY,+8,t(9;22)(q34;q11)[2] C: 46,XY,t(9;22)(q34;q11)[20], then 46,XY [14]	HHT IFN Ara-C	10	Imatinib SCT	Y	Y	Y	NO	200	Alive
#2	43/M	A: 46,XY,t(9;22)(q34;q11)[20] B: 46,XY,t(9;22)(q34;q11)[16]/47,XY,+8,t(9;22)(q34;q11)[2]/46,XY[2] C: 46,XY,t(9;22)(q34;q11.2)[1]/46,XY[19], then 46,XY[20]	Hydrea IFN Imatinib	31	Imatinib	Y	Y	Y	NO	171	Alive
#3	42/M	A: 46,XY,t(9;22)(q34;q11)[5]/46,XY[15] B: 46,XY,t(9;22)(q34;q11)[6]/47,XY,+8,t(9;22)(q34;q11)[5]/46,XY[14] C: 46,XY,t(9;22)(q34;q11)[4]/46,XY[16], then 46,XY[20]	Hydrea IFN Ara-C	13	IFN Ara-C Imatinib	Y	Y	Y	NO	167	Alive
#4	56/F	A: 46,XX,t(9;22)(q34;q11)[19]/46,XX[1] B: 46,XX,t(9;22)(q34;q11.2)[16]/47,XX,+8,t(9;22)(q34;q11.2)[2]/46,XX[2] C: 46,XX,t(9;22)(q34;q11.2)[20]	HHT IFN Ara-C Imatinib	23	Dasatinib Imatinib	N	N	Y	NO	66	Dead
#5	49/F	A: 46,XX,t(9;22)(q34;q11)[3]/46,XX[16] B: 46,XX,t(9;22)(q34;q11.2)[5]/47,XX,+8,t(9;22)(q34;q11.2)[4]/46,XX[11] C: 46,XX[20]	IFN	36	Imatinib Dasatinib	Y	Y	Y	NO	171	Alive
#6	46/F	A: 46,XX,t(9;22)(q34;q11)[20] B: 46,XX,t(9;22)(q34;q11.2)[10]/47,XX,+8,t(9;22)(q34;q11.2)[10] C: 46,XX[20]	Hydrea Imatinib	41	SCT	Y	Y	Y	NO	150	Alive
#7	44/M	A: 46,XY,t(9;22)(q34;q11.2)[14]/45,X,-Y[3]/46,XY[3] B: 46,XY,t(9;22)(q34;q11.2)[9]/47,XY,+8,t(9;22)(q34;q11.2)[11] C: N/A	Hydrea INF Ara-C Imatinib	26	Imatinib INF Ara-C	N/K	N/K	N/K	N/K	7.2	Alive
#8	40/M	A: 46,XY,t(9;22)(q34;q11)[19] B: 46,XY,t(9;22)(q34;q11.2)[12]/47,XY,+8,t(9;22)(q34;q11.2)[8] C: 46,XY,t(9;22)(q34;q11.2)[15]/46,XY,t(9;22)(q34;q11.2),i(17)(q10)[3]/47,XY,+8, t(9;22)(q34;q11.2)[1]/47,XY,+8,t(9;22)(q34;q11.2),i(17)(q10)[1], then lost cytogenetic F/U	IFN Ara-C	9	Imatinib	N/K	N/K	N/K	N/K	18.2	Dead
#9	44/M	A: 46,XY,t(9;22)(q34;q11.2)[14]/46,XY[6] B: 48,XY,+8,+8,t(9;22)(q34;q11.2)[11]/46,XY[9] C: 46,XY[20]	Hydrea Ara-C IFN Imatinib	54	Nilotinib SCT	Y	Y	Y	NO	132	Alive
#10	44/M	A: 46,XY,t(9;22)(q34;q11.2)[20] B: 46,XY,t(9;22)(q34;q11.2)[17]/47,XY,+8,t(9;22)(q34;q11.2)[3] C: 46,XY,t(9;22)(q34;q11.2)[20]	Hydrea IFN Imatinib	53	Nilotinib Decitabine	N	N	Y	Yes	35	Dead
#11	40/M	A: 47,XY,+8[1]/46,XY[9] B: 47,XY,+8,t(9;22)(q34;q11.2)[2]/46,XY[18] C: 46,XY[20]	Imatinib	73	Dasatinib Nilotinib	Y	Y	Y	NO	105	Alive
#12	28/M	A: 46,XY,t(9;22;16;16)(q34;q11.2;q12;q22)[20] B: 46,XY,t(9;22;16;16)(q34;q11.2;q12;q22)[17]/47,XY,+8,t(9;22;16;16)(q34;q11.2;q12;q22)[2]/46,XY[1] C:46,XY,t(9;22;16;16)(q34;q11.2;q12;q22)[10]/46,XY,der(9)t(9;22;16;16)(q34;q11.2;q12;q22), der(16)t(9;22;16;16),der(16)t(9;22;16;16),der(22)idic(22)(p11.2)t(9;22;16;16)[5]/46,XY[5]	Hydrea INF Imatinib	42	Imatinib Dasatinib	N	N	Y	Yes	65	Dead
#13	71/F	A: 46, XY, t(9;22) (q34;q11.2)[20] B: 46,XX,t(9;22)(q34;q11)[14]/47,XX,+8,t(9;22)(q34;q11)[6] C: 46,XX[20]	IFN	26	Imatinib	Y	Y	Y	NO	151	Alive
#14	35/F	A: 46,XX[20] B: 46,XX,t(9;22)(q34;q11.2)[18]/47,XX,+8,t(9;22)(q34;q11.2)[2] C: 46,XX,t(9;22)(q34;q11.2)[19]/46,XX[1], then 46,XX[20]	Imatinib	42	Imatinib Nilotinib SCT	Y	Y	Y	NO	22	Dead
#15	51/M	A: 46,XY,t(9;22)(q34;q11.2)[29] B: 46,XY,t(9;22)(q34;q11.2)[18]/47,XY,+8,t(9;22)(q34;q11.2)[2] C: 46,XY,t(9;22)(q34;q11.2)[20]	Hydrea IFN Ara-C Imatinib	92	Decitabine HHT Imatinib dasatinib	N	N	Y	NO	33	Dead
#16	66/M	A: 46,XY,t(9;22)(q34;q11.2)[20] B: 46,XY,t(9;22)(q34;q11.2)[12]/47,XY,+8,t(9;22)(q34;q11.2)[8] C: 46,XY,t(9;22)(q34;q11.2)[19]/47,XY,+8,t(9;22)(q34;q11.2)[1], then lost cytogenetic F/U	IFN cytarabine Imatinib	24	Imatinib	N/K	N/K	N/K	NO	85	Dead
#17	43/F	A: 46,XX,t(9;22)(q34;q11.2)[4]/46,XX[16] B: 47,XX,+8,t(9;22)(q34;q11.2)[15]/46,XX[5] C: 46,XY[20]	Hydrea Imatinib Dasatinib	98	Nilotinib SCT	Y	Y	Y	NO	48	Alive
#18	38/F	A: 46,XX[5] B: 46,XX,t(9;22)(q34;q11.2)[15]/47, +8, t(9;22)(q34;q11.2) [3]/46,XX[2] C: 46,XX[20]	Imatinib	97	Dasatinib	Y	Y	Y	NO	47	Alive
#19	28/M	A: 46,XY, t(9;22)(q34;q11.2)[20] B: 46,XY,t(9;22)(q34;q11.2)[4]/47,XY,+8,t(9;22)(q34;q11.2)[2]/46,XY[14] C: 46,XY[20]	Imatinib	3	Imatinib	Y	Y	Y	NO	128	Alive
#20	52/M	A: 46,XY,t(9;22)[19]/46, XY[1] B: 46,XY,t(9;22)(q34;q11.2)[27]/47,XY,+8,t(9;22)(q34;q11.2)[2]/50,XY,+X,+8, t(9;22)(q34;q11.2),+13,+17[1] C: 46,XY,t(9;22)(q34;q11.2)[1]/46,XY[19]	Imatinib	11	Imatinib	N	N	Y	NO	66	Alive
#21	51/F	A: 46,XX,t(9;22)(q34;q11.2)[19]/46,XX[1] B: 46,XX,t(9;22)(q34;q11.2)[10]/47,XX,+8,t(9;22)(q34;q11.2)[8] C: 47,XX,t(9;22)(q34;q11.2),+mar[20], then lost cytogenetic F/U.	IFN Imatinib Dasatinib Nilotinib	85	HHT Imatinib Bafetinib	N/K	N/K	Y	Yes	8	Dead
#22	34/F	A: 46,XX,t(9;22)(q34;q11.2)[10]/47,X,add(X)(q28),+11,del(15)(q13q15)[3]/46,XX[7] B: 47,XX,+8,t(9;22)(q34;q11.2)[13]/47,XX,+11[1]/46,XX[6] C: 46,XX,t(9;22)(q34;q11.2)[20], then lost cytogenetic F/U.	Imatinib Dasatinib	63	Bosutinib SCT	N/K	N/K	Y	NO	62	Alive
#23	48/F	A: 46,XX[20] B: 46,XX,t(9;22)(q34;q11.2)[10]/47,XX,+8,t(9;22)(q34;q11.2)[6] C: 46,XX,t(9;22)(q34;q11.2)[18]/46,XX[2], then 46,XX[20]	Imatinib	16	Imatinib Bosutinib Nilotinib	Y	Y	Y	NO	121	Alive
#24	51/M	A: 46,XY,t(9;22)(q34;q11.2)[2]/46,XY[17] B: 46,XY,t(9;22)(q34;q11.2)[6]/47,XY,+8,t(9;22)(q34;q11.2)[5]/46,XY[9] C: 46,XY,t(9;22)(q34;q11.2)[20]	Imatinib Dasatinib	42	Imatinib Bosutinib Nilotinib	N	N	Y	NO	30	Dead
#25	27/F	A: 46,XX,t(9;22)(q34;q11.2)[11] B: 46,XX,t(9;22)(q34;q11.2)[9]/47,+8,t(9;22)(q34;q11.2) [11] C: 46,XY[20]	Imatinib Nilotinib Dasatinib	21	SCT	Y	Y	Y	NO	39	Alive
#26	31/M	A: 46,XY,t(9;22)(q34;q11.2)[17]/46,XY[3] B: 46,XY,t(9;22)(q34;q11.2)[18]/47,+8,t(9;22)(q34;q11.2) [2] C: 46,XX[20]	Nilotinib Imatinib	20	Ponatinib SCT	Y	Y	Y	NO	37	Alive
#27	20/F	A: 46,XX,t(9;22)(q34;q11.2)[20] B: 46,XX,t(9;22)(q34;q11)[15]/47,XX,+8,t(9;22)(q34;q11)[5] C: N/A (lost cytogenetic F/U)	IFN Ara-C	20	N/K	N/K	N/K	N/K	N/K	126	Dead
#28	70/F	A: 46,XX,t(9;22)(q34;q11.2)[20] B: 46,XX,t(9;22)(q34;q11)[18]/47,XX,+8,t(9;22)(q34;q1*1)[2]* C: N/A(lost cytogenetic F/U)	Hydrea IFN	10	N/K	N/K	N/K	N/K	N/K	4	Alive

Abbreviations: BP, blast phase; CCyR, complete cytogenetic response; HHT, homoharringtonine; IFN, interferon; MMR, major molecular response; N/A, not available SCT, stem cell transplant.

Age, age of CML diagnosis; Karyotype, A (karyotype before+8 emergence), B (karyotype at the time of +8 emergence) and C (representative karyotype after +8 emergence); Before, the treatment before +8 emergence; Interval-1, the interval from the initial CML diagnosis to +8 emergence; After, the treatment after +8 emergence; Interval-2, the interval from the time of +8 emergence to the last follow-up; N/K, not known. The patients lost follow-up or had too short follow-up time for evaluation of cytogenetic response.
